# What Do We Mean by Behavioral Disinhibition in Frontotemporal Dementia?

**DOI:** 10.3389/fneur.2021.707799

**Published:** 2021-07-07

**Authors:** Nahuel Magrath Guimet, Bruce L. Miller, Ricardo F. Allegri, Katherine P. Rankin

**Affiliations:** ^1^Atlantic Fellow for Equity in Brain Health at the Global Brain Health Institute, University of California, San Francisco, San Francisco, CA, United States; ^2^Department of Cognitive Neurology, Neuropsychiatry and Neuropsychology, Instituto Neurológico Fleni, Buenos Aires, Argentina; ^3^Global Brain Health Institute, University of California, San Francisco, San Francisco, CA, United States; ^4^Memory and Aging Center, Department of Neurology, University of California, San Francisco, San Francisco, CA, United States; ^5^Department of Neurosciences, Universidad de la Costa (CUC), Barranquilla, Colombia

**Keywords:** disinhibition, semantic variant primary progressive aphasia, brain networks, semantic cognition, frontotemporal dementia, behavioral variant frontotemporal dementia, behavioral disinhibition

## Abstract

Behavioral variant frontotemporal dementia, unlike other forms of dementia, is primarily characterized by changes in behavior, personality, and language, with disinhibition being one of its core symptoms. However, because there is no single definition that captures the totality of behavioral symptoms observed in these patients, disinhibition is an umbrella term used to encompass socially disruptive or morally unacceptable behaviors that may arise from distinct neural etiologies. This paper aims to review the current knowledge about behavioral disinhibition in this syndrome, considering the cultural factors related to our perception of behavior, the importance of phenomenological interpretation, neuroanatomy, the brain networks involved and, finally, a new neuroscientific theory that offers a conceptual framework for understanding the diverse components of behavioral disinhibition in this neurodegenerative disorder.

## Introduction

Human behavior is complex and results from the interaction of psychological, social, cultural, and biological factors. Furthermore, we know that specific brain structures play a leading role in directing behavior, as evidenced by the social behavior disorders that occur after events that directly or indirectly affect the brain. Among these structures, the prefrontal cortex has a central role (1).

Behavioral variant frontotemporal dementia (bvFTD) is a neurodegenerative clinical syndrome that affects the frontal and temporal lobes and is characterized by personality and behavior changes. These changes include apathy, loss of empathy, disinhibition, compulsive/ritualistic behavior, and hyperorality, often overlapping with one another (2, 3). Of the above, behavioral disinhibition is one of the most frequent and distinctive symptoms (2, 4). Yet there is no single definition of disinhibition that encompasses the vast number of behaviors that could be labeled as such. Thus, the concept of “behavioral disinhibition” becomes an umbrella term associated with a myriad of clinical presentations.

Much emphasis has been placed on discriminating frontotemporal dementia (FTD) from other neurodegenerative diseases, primarily Alzheimer's disease, as there may be symptomatic overlap (5–7), but because bvFTD is a disorder of behavior changes, one of the main diagnostic challenges is to differentiate bvFTD from primary psychiatric disorders (PPD) (8–10). Of the many psychiatric disorders that overlap syndromically with bvFTD, bipolar disorder and schizophrenia are uniquely problematic (11). This can lead to a significant delay in diagnosis, increasing the stress that this disease generates for patients and family members.

Behavioral disinhibition is a complex phenomenon that can arise as a result of cognitive deficits in different domains and not only due to a loss of inhibition. This paper aims to review the current knowledge about this symptom, considering the cultural factors related to our perception of behavior, the importance of phenomenological interpretation, neuroanatomy, the brain networks involved and, finally, a new neuroscientific theory that offers a conceptual framework for understanding behavioral disinhibition in bvFTD and related FTD syndromes.

## What Is Behavioral Disinhibition?

As previously mentioned, there is no single, universally accepted conception of “behavioral disinhibition.” Definitions often used point to the manifestation of socially disruptive or morally unacceptable behaviors (12). Current diagnostic criteria for bvFTD describe that behavioral disinhibition may manifest as “socially inappropriate behavior,” “loss of manners/decorum,” or “impulsive, rash or careless actions” (2). While this description provides a framework for clinical interpretation, certain behaviors may be controversial when considering them as a symptom of the disease. Of the vast number of factors that may condition our interpretation of behavioral phenomena, two components are of particular importance. First, premorbid psychological factors should be probed to determine whether the problematic behavior is new or longstanding. One of the characteristics of bvFTD is that the behavioral changes emerge as a result of frontotemporal lobar degeneration (FTLD) pathology, thus at the time of disease onset there is a marked change in the behavioral pattern compared to a previous, premorbid status. By contrast, many patients with PPD may also exhibit behaviors that are interpreted as inappropriate, but these are intrinsic to their usual conduct, meaning that there has not been a marked change in the behavioral pattern. On the other hand, we now know that many FTLD gene mutation carriers present with psychiatric manifestations years before meeting criteria for a bvFTD diagnosis (10, 13, 14), making this distinction of timing less definitively diagnostic.

Second, cultural factors are important to consider, and the clinician must always ask the question whether or not the behavior atypical for that person's cultural background. Social conventions, a product of a community's history and cultural traditions, may be seen as inappropriate or bizarre from the perspective of another cultural paradigm. Some of these behaviors are so far from the norm that they are easily interpreted as a foreign cultural practice in the eyes of the observer. For example, when seeing a person in San Francisco wearing the ceremonial clothing of an Andean aboriginal community, one assumes that this is someone from another culture rather than someone who is breaking social norms. Sometimes these cultural differences are more subtle, however, and can lead to misinterpreting a behavior as pathological. For example, in Latin America, it is common to salute one another with a kiss or hug, even if there is no great familiarity between individuals, while this conduct may be seen as highly inappropriate in an Anglo-Saxon society such as the U.S. or the U.K. As these examples highlight, there are individual and cultural aspects that shape which acts are interpreted as socially inappropriate or disinhibited.

## The Importance of Phenomenology

Because human behavior is potentially boundless in its manifestations and differs enormously among subjects, clinicians have historically attempted to categorize these behaviors to study them phenomenologically. One objective for carefully classifying the observed phenomena is to enable a search for the causes, and the underlying biological mechanisms, that produce these behaviors. An example of this process is the description made by Marin in 1991 of apathy, describing in his first paper 3 types of apathy (behavioral, cognitive, and affective) (15). With the advance of new neuroimaging techniques and deeper knowledge of the neuropsychological processes these categories changed over time (16–20). At present, Radakovic's classification for apathy contemplates 3 categories (initiation, executive, and emotional) and he developed the dimensional apathy scale (DAS) to differentiate them (21).

Much of the information currently available on neuropsychiatric symptoms in dementia, and the phenomenology of disinhibition in particular, comes from research conducted in recent years using the Neuropsychiatric Inventory (NPI), one of the most commonly used scales in the dementia field (1, 11, 22–24). The NPI is frequently employed for the detection of behavioral symptoms in dementia as it assesses several symptomatic domains at once. Yet scales as broad as this one may fail to differentiate among real-life situations that could be categorized as disinhibition (7, 25, 26).To address this, and conduct a more thorough study of disinhibition, some studies use multiple scales simultaneously (7), and may further break down the symptom into various subcategories through principal component analysis (26, 27). Although these strategies offer a broader assessment of behavioral symptoms, there is still no consensus on how to classify disinhibited behavior to overcome the important limitations described above.

Other behavioral scales that are also used to objectively assess disinhibition in dementia patients include the Frontal Assessment Battery (FAB) (28), the Behavioral Inhibition Scale (BIS/BAS) (29), and the Frontotemporal Dementia Rating Scale (FRS) (30). These scales measure behavior either through the clinician's assessment (e.g., by performing specific tests or by qualitatively rating behavior), or through data provided by a family member or caregiver informant. As previously established, however, psychological factors and cultural differences may impact our assessment of the patient's behavior, affecting which behaviors each measure labels as disinhibited. Because of this, contextual information provided by informants can help to bridge this cultural barrier.

In an attempt to explore the phenomena behind behavioral disinhibition in FTD, Paholpak et al. (26) used the Frontal System Behavioral Scale (FrSBe) to subcategorize it into two modalities: (1) disinhibition related to the transgression of social norms and personal boundaries, which they called “person-based disinhibition,” and (2) disinhibition linked to the inability to refrain behavior, which they categorized as “impulsivity.” With similar results, an ecological study by Godefroy and Tanguy evaluated the reactions of 17 bvFTD patients with disinhibited behaviors simulating real-life situations, and they were able to differentiate a group with social disinhibition and another with a mixture of impulsivity and compulsivity (31). Thus, similar to the previous work done in the phenomenology of apathy, new ways of classifying disinhibited behavior may allow us to better identify the underlying mechanisms involved in bvFTD.

## The Evolving Concept of Behavioral Disinhibition

The classical neuroanatomical conception of behavioral disinhibition arises from the premise that there are brain structures that generate impulses or actions that the individual wishes to perform, and these, when they could be construed as socially inappropriate or disadvantageous, are inhibited by the frontal lobe (12, 32). Thus, there are at least two mechanisms by which disruptive behavior may arise. First, there may be a compromise of the frontal structures responsible for inhibiting the impulse (i.e., “loss of brakes”), or there may be a hyperactivation of the structures that generate the impulse (i.e., “excess gas”). This inhibitory model has its roots in the mid-nineteenth century in studies of motor function, when it was noted that the motor cortex exerts inhibitory control over spinal reflex arcs. From this discovery, Ferrier, observing that lesions in the prefrontal cortex (PFC) of monkeys caused behavioral changes, hypothesized that the PFC has an inhibitory function on behavior (12). This model was reinforced by the famous Phineas Gage behavioral disinhibition case, in which a massive lesion in the left PFC caused the behavioral changes Harlow described as “fitful, irreverent, indulging at times in the grossest profanity (which was not previously his custom), manifesting but little deference for his fellows, impatient of restraint or advice when it conflicts with his desires, at times pertinaciously obstinate, yet capricious and vacillating, devising many plans of future operation, which are no sooner arranged that they are abandoned in turn for others appearing more feasible” (33).

Clinical cases of behavioral disinhibition, such as Phineas Gage's, laid the groundwork for the lesion-based studies that led to the emergence of the modular model of brain functioning, which posits that specialized processing is performed by well-defined brain regions. Under this model, when studying behavioral disinhibition in FTD syndromes, several studies found similar patterns of brain involvement implicating the OFC (34–37) and right anterior temporal lobe (ATL) (1, 36–38). Nonetheless, there are discrepancies among studies. For example, some papers demonstrated involvement of the striatum in relation to disinhibition (1, 38), while others related it to symptoms such as apathy and eating disorders (34). Something similar occurs with the anterior cingulate cortex (ACC), where some authors relate it to behavioral disinhibition (36, 37), while others highlight its relationship to apathy (1, 34, 35). One possible explanation for these discrepancies is that different aspects of the same symptom are included under the broad concept of behavioral disinhibition, but these variants have different anatomical correlates. Support for this is found in the previously cited work of Paholpak et al. that subclassifies behavioral disinhibition into person-based and impulsive components. In analyzing the neural correlates, they found that person-based disinhibition correlated with the left superior temporal sulcus; whereas impulsivity was more closely related to changes in the right orbitofrontal cortex (OFC) (21).

As computational brain imaging techniques have evolved, another framework for understanding neural functions has arisen to complement and enhance structural explanations. In the connectivity model, cognitive processes, which result in social or moral behavior, are a consequence of evolutionary pressures that have shaped the brain circuits that structure emotion, motivation, and social cognition (39, 40). We now know that inhibitory control involves a set of complex cognitive processes that operate online and in synchrony, evaluating and modulating the response to external stimuli (25, 41, 42). Advances in functional neuroimaging have identified the intrinsically connected networks (ICNs) that form these neural circuits.

ICNs are a set of large-scale functionally connected brain networks that form the organizational elements of the brain's architecture (43–45). ICNs offer insight into the way in which cognition is performed by sets of structures organized into distinct modular systems. Each subsumes a different higher-order cognitive function that is more complex than any one structure can perform alone, such as grammar sequencing, controlled visual search, or salience-driven attention. Some ICNs are selectively vulnerable to FTLD neuropathology and therefore are particularly compromised in bvFTD, and these are central to understanding the phenomena of behavioral disinhibition (46). These ICNs are the salience network (SN), the semantic appraisal network (SAN), and the task control networks (47, 48).

The SN is related to socioemotional processing because it is responsible for the assessment of internal and external stimuli that are particularly salient for the individual. This network has two main cortical hubs in the ventral anterior insula and ACC, as well as several subcortical nodes (amygdala, hypothalamus, dorsomedial thalamus, and periaqueductal gray matter) (48, 49). In both the ACC and the frontoinsular cortex there are Von Economo neurons, which have been attributed a central role in social cognition. These neurons are uniquely part of the SN and their dysfunction is proposed to be a driver of bvFTD (50). Degree of intrinsic connectivity in the SN has been directly linked to socioemotional sensitivity (51), a central component of social cognition that allows for adequate alertness to social cues. Thus, dysfunction of this network can lead to failure to recognize negative reinforcers, such as punishment signals, that inhibit us from socially inappropriate behavior, which may in turn lead to behavioral disinhibition.

Another network closely related to socioemotional processing is the SAN. This network plays a central role in comprehending emotions and automatically assigning emotional valence to stimuli so that the SN can then recognize their personal salience (48). Thus, the SAN is key in correctly guiding behavior toward reward and away from punishment, and its dysfunction is associated with semantic deficits, and therefore errors in evaluation of potential outcomes, that may contribute to behavioral disinhibition (52). This network has its hub in the dorsomedial anterior part of the temporal lobe and has nodes in the subgenual cingulate, the head of the caudate, nucleus accumbens, amygdala, and cerebellum (48).

The third mechanism of paramount importance for understanding behavioral disinhibition in bvFTD are the ICNs related to task control. Dosenbach et al. (53) describe two networks whose activity is oriented to the adaptive and stable aspects of task control. The frontoparietal network, linked to adaptive task initiation and adjustment of control in response to feedback, has nodes in the intraparietal sulcus, dorsolateral prefrontal cortex, inferior parietal lobe, precuneus, and midcingulate cortex. The cinguloopercular network, related to the stable maintenance of resources necessary to carry out an operation, consists of dorsal anterior cingulate/medial superior frontal cortex, anterior insula/frontal operculum, anterior prefrontal cortex and thalamus (53). Although both networks function in parallel, the frontoparietal network seems to be crucial for selecting and initiating online control processes that inhibit behavior, while the cinguloopercular circuit is central in focusing attention on maintaining inhibition for the duration of the task. Thus, dysfunction of either circuit may lead to behavioral disinhibition.

## Toward a New Paradigm?

Behavioral disinhibition, being strongly associated with the disruption of social norms is traditionally studied through the paradigms of social cognition, however, there are reasons to think that this phenomenon is more fundamentally related to neuroscience models of language and object knowledge (48). In this line of research, Lambon Ralph et al. proposed the controlled semantic cognition (CSC) model (54), in which the term “semantic cognition” describes a set of supramodal verbal and non-verbal processes that underpin how meaning is structured from the environment, including but not limited to, social environments. Under the CSC paradigm, two interrelated systems subsume semantic cognition: the representational system and the process control system.

The representational system is related to the acquisition and long-term storage of conceptual knowledge. This system has a “hub-and-spoke” architecture in the brain, where the modality-specific processing systems (“spoke nodes”; e.g., audition, face-processing, valence, etc.) provide the blocks of sensory, motor, linguistic, and affective information to build concepts. A particular feature of this system is that it proposes the existence of a supramodal “hub,” located bilaterally in the ATL, which is responsible for integrating the incoming transmodal information from each spoke and encoding it at a more abstract level of representation. The connection between the hub and the spokes is bidirectional, and knowledge conceptualization emerges from joint processing across the levels of this representational system.

The second system of this model, the process control system, is responsible for directing conceptual knowledge to produce an operation. The logic behind this mechanism is that it is not necessary to access all the information that exists about an object to make decisions about it or operate on it. Thus, this control system guides the efficient and fast retrieval of only the most practically relevant information out of the representational “library” to enable decisions and action in real-time. Anatomically, this control system is located bilaterally in the ventrolateral prefrontal and temporoparietal cortex.

**Figure 1:**
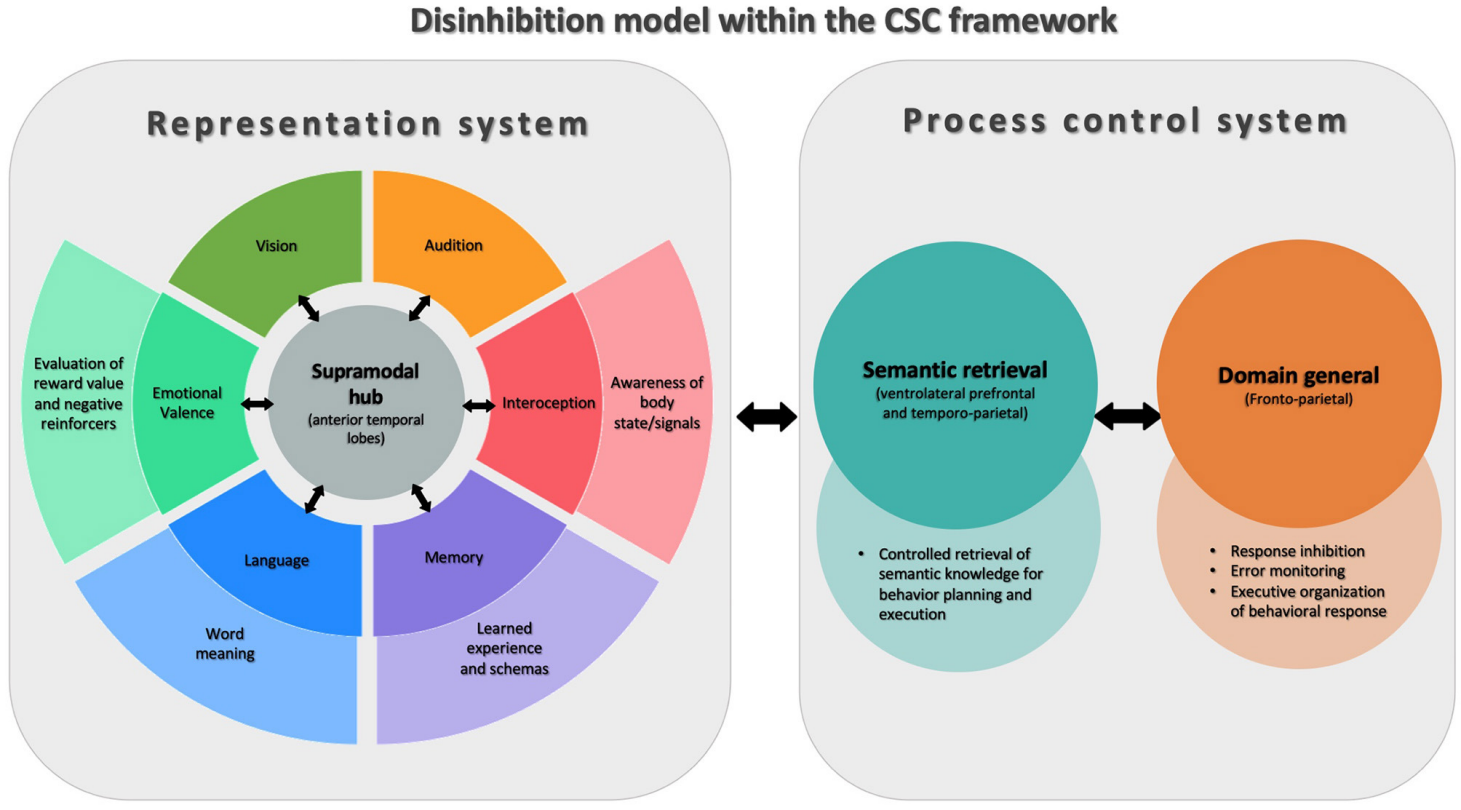
Model of disinhibition conceptualized via Controlled Semantic Cognition theory (CSC). This figure shows the two interconnected systems that are part of the CSC theory. On the left of the figure is the representational system, whose function is to acquire and store conceptual knowledge. For this, in the center, there is a supramodal semantic hub (anterior temporal lobes) that receives modality-specific information from different systems (“spokes”) throughout the brain. On the right is the process control system, involved in the successful application of conceptual knowledge, composed of semantic retrieval and general domain processes. The figure shows how components of the CSC system support different aspects of cognition that are involved in behavioral inhibition and disinhibition.

The CSC paradigm provides a framework for understanding the acquisition, consolidation, and evocation of conceptual knowledge regardless of its modal source. Recently, Binney and Ramsey proposed that by bridging socioemotional processing, language and behavior, this model is especially relevant to social cognition (55). This paradigm may be of particular interest for understanding symptoms in FTD like behavioral disinhibition (Figure 1). The impairment of the representational system may lead to the loss of the knowledge necessary to recognize, understand, and evaluate social rules and, thus, to prevent inappropriate behaviors. On the other hand, dysfunction of the process control system may compromise the executive mechanisms necessary to prevent impulsive, inattentive, or disorganized behavior choices. Evidence in favor of this is provided by the example of semantic variant of primary progressive aphasia (svPPA), a variant of FTD, which primarily affects semantic knowledge and particularly involves the ATL. Importantly, svPPA is associated with the early appearance of major neuropsychiatric symptoms, behavioral disinhibition being one of the most frequent (56, 57). Moreover, in other forms of FTD such as non-fluent primary progressive aphasia (nfvPPA), with more frontal than temporal involvement, milder disinhibition that is predominantly related to impulsivity can often be found (27, 58).

Models such as the CSC seem particularly interesting to understand the underlying neurobiological processes in bvFTD since, it seems to respect and unify several of the previous findings. Thus, when faced with disinhibited behavior in a patient with bvFTD, it is possible to conjecture from the type of disinhibition (person-based or impulsivity), which brain regions are affected, which ICNs are involved, and which component of the CSC model the behavior corresponds to Table 1.

**Table 1:** Correlation model between clinical scenarios and interpretations of different conceptual frameworks.

**Real life situation example**	**Clinical interpretation**	**Disinhibition phenotype**	**Main brain regions affected**	**Brain network involved (ICN)**	**Controlled semantic cognition system (CSC)**
Patient stops to initiate conversations with strangers in public places and asks about private matters.	The patient does not understand that it is socially inappropriate to ask about private matters to strangers. May correspond with loss of knowledge of social norms and expectations.	Person-based	Subgenual cingulate cortex. Anterior temporal lobe.	Semantic appraisal network (SAN)	Representation
Male patient enters the women's restroom at his place of employment because he is attracted to a female colleague and wanted to see her. When the situation is brought to his attention, he understands that this behavior is socially inappropriate, however, he repeats it.	The patient understands and knows that the act is inappropriate/immoral in nature, however, when confronted with the situation this does not resonate emotionally with them and performs the action anyway. May correspond with loss of sensitivity to punishment cues.	Person-based	Ventrolateral prefrontal cortex. Ventral anterior insula	Salience network (SN)	Representation
Patient enters a store, sees an object he wants and takes it without paying for it. When questioned about this, he says that he knows it is wrong and feels guilty about it.	Patient understands the situation, it resonates on an emotional level, but nevertheless they cannot stop the action or fails to analyze the cost/benefit of the action.	Impulsivity	Intraparietal sulcus. Dorsolateral prefrontal cortex.	Fronto-parietal network	Control
During the clinical interview the patient seems distracted, gets up from the seat, changes the topic of conversation, asks constantly if he/she can leave now even though he/she does not seem anxious or to be discussing a disturbing topic.	Patient understands the situation he/she is, but cannot sustain the resources to maintain conversation or behavior for a prolonged period of time. May correspond with cognitive impersistence or motor restlessness.	Impulsivity	Dorsal anterior cingulate cortex. Middle frontal gyrus. Frontal operculum. Caudate.	Cinguloopercular network	Control

## Discussion and Conclusions

Behavioral disinhibition is one of the most prominent and disturbing manifestations of bvFTD. However, its interpretation and analysis is a complex task, thus the phenomenon has multiple edges and challenges for its study. One of the first limitations we encounter is that many of the instruments we use to objectively evaluate behavioral disinhibition are imprecise. Our measurement of the symptom is only as specific as the instruments we use. Thus, scales that assess disinhibition globally, such as the NPI, do not capture the spectrum of manifestations associated with behavioral disinhibition, or provide information on the key neural contributors to the observed behavior. Ideally, and following the example cited on apathy and its subcategories, a scale for behavioral disinhibition should be able to capture the subtype of deficit seen in the patient, such as whether the phenomenon we observe is due to a lack of understanding of social norms, a loss of impulse control, or both.

Numerous hypotheses attempting to elucidate the causes behind behavioral disinhibition have emerged, and they have evolved from their original conceptions at the end of the nineteenth century to the present day. The first lesion-based models, which led to modular localizationist theories, have culminated in the current functional connectivity model, where cognition is the result of complex interactions among different hubs connected through ICNs. Among these intrinsic brain networks, some seem to be particularly affected in bvFTD, such as the SN, SAN, and networks involved in task control, and thus appear to be directly related to these patients' behavioral disinhibition syndromes.

With a phenomenological perspective, some authors have created subcategories of behavioral disinhibition to be able to better study the neural processes linked to this behavior. Thus, it is possible to find at least two types of behavioral disinhibition, a person-based etiology, with greater involvement of the ATL and OFC; and a version of disinhibition closely related to impulsivity, with greater dorsal PFC involvement. Considering that both the ATL and OFC are hubs of the SAN, we believe that this network is of paramount importance to better understand the person-based mechanisms leading to behavioral disinhibition, while the adaptive and stable task control networks comprising dorsomedial and dorsolateral frontoparietal regions appear to be particularly important for behavioral control and management of impulsivity.

In the past decade, neuroscientific accounts of behavior have matured and flourished, and insights from this domain can be highly relevant and provide a more nuanced understanding of patients' symptoms. The distinct interrelated systems for representation and control in the CSC model provide a useful framework for understanding various aspects of behavioral disinhibition in FTD. The impairment of the representational system may explain the occurrence of socially inappropriate behavior due to the loss of semantic knowledge of social norms or the compromise of the emotional valence attached to such information. In turn, deficits in the process control system may explain how patients' behaviors may became disinhibited through impairment of the online executive task control system.

Throughout this paper, we have reviewed the existing barriers to diagnosing and interpreting the phenomena associated with what we understand as behavioral disinhibition. Despite these limitations, important advances have been made toward identifying key processes and structures involved in the genesis of this complex symptom. In this way, it is clear that progress in the neuropsychiatry of disinhibition can only arise through greater collaboration with other disciplines, including by incorporating novel imaging methods and neuroscientific models to refine our theories and enhance our discoveries.

## Author Contributions

NM and KR contributed to the conception and design of the manuscript. NM wrote the first draft. KR was the primary reviewer of the draft. All authors contributed to manuscript revision, read, and approved the submitted version.

## Conflict of Interest

The authors declare that the research was conducted in the absence of any commercial or financial relationships that could be construed as a potential conflict of interest.
